# Two Cathepsins B Are Responsible for the Yolk Protein Hydrolysis in *Culex quinquefasciatus*

**DOI:** 10.1371/journal.pone.0118736

**Published:** 2015-02-24

**Authors:** Alexandre S. Moura, André F. Cardoso, André L. Costa-da-Silva, Carlos E. Winter, A. Tania Bijovsky

**Affiliations:** 1 Departamento de Parasitologia, Instituto de Ciências Biomédicas, Universidade de São Paulo, São Paulo, SP, Brasil; 2 Instituto Nacional de Ciência e Tecnologia em Entomologia Molecular, INCT-EM, Rio de Janeiro, RJ, Brasil; Universidade Federal do Rio de Janeiro, BRAZIL

## Abstract

Despite the established role of *Culex quinquefasciatus* as a vector of various neurotropic viruses, such as the Rift Valley and West Nile viruses, as well as lymphatic filariasis, little is known regarding the organism’s reproductive physiology. As in other oviparous animals, vitellogenin, the most important source of nutrients for the embryo development, is digested by intracellular proteases. Using mass spectrometry, we have identified two cathepsin B homologues partially purified by self-proteolysis of *Cx. quinquefasciatus* total egg extract. The transcriptional profile of these two cathepsin B homologues was determined by quantitative RT-PCR, and the enzymatic activity associated with the peptidase was determined in ovaries after female engorgement. According to the VectorBase (vectorbase.org) annotation, both cathepsin B homologues shared approximately 66% identity in their amino acid sequences. The two cathepsin B genes are expressed simultaneously in the fat body of the vitellogenic females, and enzymatic activity was detected within the ovaries, suggesting an extra-ovarian origin. Similar to the transcriptional profile of vitellogenin, cathepsin B transcripts were shown to accumulate post-blood meal and reached their highest expression at 36 h PBM. However, while vitellogenin expression decreased drastically at 48 h PBM, the expression of the cathepsins increased until 84 h PBM, at which time the females of our colony were ready for oviposition. The similarity between their transcriptional profiles strongly suggests a role for the cathepsin B homologues in vitellin degradation.

## Introduction

*Culex quinquefasciatus* (Diptera: Culicidae) is a cosmopolitan mosquito that is highly anthropophilic and completely adapted to urban conditions. This mosquito is a competent vector of neurotrophic viruses such as the St. Louis and Japanese encephalitis viruses, the eastern and western equine encephalomyelitis viruses and the Rift Valley and West Nile viruses [1–4]. Moreover, *Cx. quinquefasciatus* is the most important Brazilian vector of *Wuchereria bancrofti*, the lymphatic filariasis agent [5].

Similar to other oviparous animals, *Cx. quinquefasciatus* generates and stores within the oocytes the nutrients needed for the embryonic development. Nutrient reserves are synthesised in the maternal fat body, a tissue analogous in function to the vertebrate liver. The primary source of amino acids and lipids for embryonic development is vitellogenin (Vg), a glycosylated phospholipoprotein that is secreted into the haemolymph and then incorporated via receptor mediated endocytosis by the developing ovarian follicles [6] and stored into the yolk platelets as vitellin [7].

The use of yolk protein as a nutrient reserve involves enzyme-mediated hydrolysis, a process that has been described to depend on various enzymes in different insect orders. Among these, the most frequently reported enzymes are cysteine proteinases, which have been described in Diptera: *Drosophila melanogaster* [8], *Musca domestica* [9,10] and *Aedes aegypti* [11,12]; Lepidoptera: *Bombyx mori* [13–18] and *Helicoverpa armigera* [19–21]; Dictyoptera: *Blatella germanica* [22] and *Periplaneta americana* [23]. While previous works in *Culex pipiens pallens* [24,25] have implicated cathepsins B and L in the atretic process of ovarian follicles degrading not only yolk proteins but also the follicular structure itself, it remains unclear whether these enzymes are required for yolk protein degradation during embryogenesis.

Haematophagous mosquitoes of the *Aedes, Anopheles* and *Culex* genera share multiple biochemical, morphological, developmental and behavioural characteristics. However, *Cx. quinquefasciatus* diverges from mosquitoes of other genera in the fine structure of their salivary glands, saliva composition [26,27], cellular and biochemical mechanisms governing blood digestion and haem detoxification [28,29] and their response to odorants and biting behaviour [30].

In the following study, we build on our initial description of the morphofunctional aspects of *Cx. quinquefasciatus* oogenesis and identify two cathepsin B proteinases, which are present in the Cx. *quinquefasciatus* eggs, expressed in the female fat bodies following a blood meal and are involved in promoting yolk protein degradation.

## Materials and Methods

### Ethics Statement

The protocols used in this work were approved by the Animal Experimentation Ethics Committee of the Institute of Biomedical Sciences (University of São Paulo, São Paulo, Brazil—process number CEAU 103/2012).

### Animals

*Cx. quinquefasciatus* (PIN strain) [31] mosquitoes were raised at 27°C, with 70–80% relative humidity and a photoperiod of 12 h dark-12 h light. Larvae were fed with ground fish food (Seravipan, Germany), and adults were fed *ad libitum* on 10% sucrose solution. As needed, 4–5 day-old adult females were fed on Balb/c mice anaesthetised with 0.3 mg/kg of xylazine hydrochloride (Calmiun, Agner União, Brazil) plus 30 μg/kg of acepromazine (Acepran, Univet S.A., Brazil).

### Egg extract

Approximately 1,500 eggs (dark eggs, harvested 24 h after oviposition) were ground with a Pellet Pestle Motor (Kontes, USA) in ice bath in a microcentrifuge tube in 200 μl of 10 mM sodium acetate buffer pH 5.0. Following centrifugation at 10,000 x*g* for 5 s, the supernatant was transferred to a new tube and the pellet was resuspended in 200 μl of sodium acetate buffer, mixed, and centrifuged, after which the supernatants were combined to obtain a final volume of 400 μl. The total protein concentration was estimated according to Bradford [32] using BSA protein as the standard.

Alternatively, white (harvested 2 h after oviposition) or dark eggs were ground as described above in a microcentrifuge tube in 200 μl of PBS pH 7.0 containing 50 μM E-64 and 1 μl/ml of a cocktail of protease inhibitors (50 μg/ml leupeptin, 5 μg/ml pepstatin, 5 μg/ml chymostatin, 5 μg/m; antipain, 5 μg/ml PMSF).

All extracts were immediately used or stored at -20°C until needed.

### Ovary extract

Ovaries of adult females 96 and 120 hours post blood meal (PBM) were processed as described above for white eggs.

### Determination of cathepsin B activity

Total protein from egg extract (40 ng) was incubated for 30 min at 27°C with 20 μM of Z-Arg-Arg-NHMec (benzyloxycarbonylarginyl-arginine 4-methylcoumarin-7-amide) in 10 mM sodium acetate buffer pH 5.0 at a final volume of 200 μl. The fluorescent product was detected using a Fluoroskan Ascent Microplate Fluorometer (Thermo Scientific, USA) with an excitation wavelength of 360 nm and emission of 450 nm. Control samples were established by adding 50 μM E-64 (N-[N-(L-3-trans-carboxyirane-2-carbonyl)-L-leucyl]-agmatine), a specific inhibitor of cysteine proteases [33]. A standard curve was obtained by measuring the fluorescence of 0.8 nM 7-amino-4-methyl Coumarin solution (AMC). The cathepsin proteolytic activities were expressed in units (U) defined as the amount of enzyme that hydrolyses 1 μmol of substrate per minute.

### Enzyme partial purification

Total egg extract was incubated at 27°C for 18 h in 10 mM sodium acetate buffer pH 5.0 supplemented with 0.5 mM phenylthiocarbamide. The degradation profile was analysed by SDS-PAGE and the enzymatic activity of the proteolytic product was determined as described above.

### Inhibition of proteolytic activity

The following protease inhibitors were added to the total egg extract to evaluate the inhibition of the reaction: 50 μM E-64 (cysteine proteases inhibitor), 1 mM PMSF (phenylmethanesulfonyl fluoride; serine proteases inhibitor), 2 mM EDTA (ethylenediamine tetraacetic acid; metalloproteases inhibitor), 10 mM pepstatin (aspartyl proteases inhibitor), and 1 mM CA-074 (N.(L-3.trans-propylcarbamoyloxirane-2-carbonyl)-L-isoleucyl-L-proline; a specific inhibitor of cathepsin B) [34]. Control reactions were incubated without protease inhibitors. The proteolytic products were analysed by SDS-PAGE, and their enzymatic activity was measured as described above.

### Sodium dodecyl sulphate polyacrylamide gel electrophoresis (SDS-PAGE)

Total egg extract and proteolytic products were mixed with an equal volume of 2x sample buffer (62 mM Tris, 50 mM DTT, 0.2% SDS, 10% glycerol, and 0.01% bromophenol blue). Proteins were resolved by SDS-PAGE using a 12% gel [35] and visualised by staining with 0.2% Coomassie Brilliant Blue R250 (w/v) dissolved in ethanol, acetic acid, and water (45:10:45, v/v/v) (Bio-Rad Laboratories, Brazil), and the molecular masses were estimated using the following protein standards: phosphorylase B (101.4 kDa), bovine serum albumin (87.5 kDa), ovalbumin (52.7 kDa), carbonic anhydrase (35.8 kDa), soybean trypsin inhibitor (22.8 kDa), and lysozyme (18.8 kDa) (Bio-Rad Laboratories, USA).

Total ovary extracts were resolved using 8% SDS-PAGE [35] by the same process described above, and molecular masses were estimated using the following protein standards: myosin (202.8 kDa), β-galactosidase (115.5 kDa), bovine serum albumin (98.2 kDa), ovalbumin (51.4 kDa) (Bio-Rad Laboratories, USA).

### Mass spectrometry analyses

Protein bands of interest were excised from the gel and in-gel digestion was performed as previously described [36]. Following desalting with Zip-Tip (Millipore, USA), tryptic fragments were analysed using a nano-HPLC-MS/MS (LCQDuo ion trap mass spectrometer; Finnigan, Thermo Fisher Scientific Inc. Waltham, Massachusetts, USA) through a 120 min gradient from 5% to 56% acetonitrile in 0.2% formic acid. The digestion spectra were analysed with the Sequest program (Thermo Fisher Scientific Inc.) using a NCBI (National Center for Biotechnology Information, http://blast.ncbi.nlm.nih.gov/blast) non-redundant database of *Culex* and confirmed with the specific vector database (https://www.vectorbase.org/). Peptides were validated using protein probability ≤ 1 x 10^–7^, dCN ≥ 0.05, and Xcorr values of 1.5, 2.2, and 2.7 for singly, doubly or triply charged peptides, respectively. Proteins with a minimum of two validated peptides were considered.

### Oligonucleotide design

Primers for RT-PCR amplification, detection and quantification of rp49 ribosomal protein and vitellogenin transcripts were designed using the Primer3 program (http://frodo.wi.mit.edu/) [37], and primers for enzyme-coding transcripts were manually designed over the 3’ noncoding region (3’UTR) of each transcript. All primers were designed using the transcript sequences available at VectorBase as template (Table 1) and synthesised by Exxtend (Brazil). The amplification efficiency for each primer was: RP49: 90,8%; CatB1: 96,82%; CatB2: 100,84%; and Vg: 101,03%.

**Table 1:** Primers designed for the detection and quantification of *Cx. quinquefasciatus* transcripts.

Gene	Access number (VectorBase)	Primers
Ribosomal protein 49 (rp49)	CPIJ001220	F- 5’ AGGTATCGACAACCGAGTGC 3’
		R- 5’ ACAATCAGCTTGCGCTTCTT 3’
Vitellogenin	CPIJ010190	F- 5’ CGTATGCCCGTAACTGGACT 3’
		R- 5’ ACTGGCAGAAGCGTTCAGAT 3’
Cathepsin B (CatB1)	CPIJ015761	F- 5’ TGGGGTGAGGACTGG 3’
		R-5’ CTGGTTGATTTTAATGAGCTGTATTTT 3’
Cathepsin B (CatB2)	CPIJ015762	F- 5’ TGGGGTGAGGACTGG 3’
		R- 5’ CTTCAGCACTTCTTTATTATGCCC 3’

### RNA extraction

Total RNA was extracted using Trizol (Life Technologies, USA) from dissected fat bodies of adult females fed on sucrose (SUC) and at 12, 24, 36, 48, 60, 72, and 84 hours post blood meal (PBM). Alternatively, whole adult females 24 hours PBM were dissected at different times and total RNA was extracted as described above. In all samples, residual genomic DNA was removed by incubation with DNase I, Amp Grade (Invitrogen, USA) and RNA integrity was evaluated by agarose gel electrophoresis. RNA was quantified using a NanoDrop ND-1000 spectrophotometer (Thermo Scientific, USA).

### RT-PCR

Reverse transcription (RT) was carried out with 2.2 μg of total RNA primed with 500 ng of Oligo dT (Life Technologies, USA) using the SuperScript II first-strand synthesis system (Life Technologies, USA) according to the manufacturer’s instructions.

PCR was performed with 1 μl of complementary DNA (cDNA) as template and 0.4 μM of each primer (Table 1). Reactions were performed in a T3 Biometra (Biometra, Germany) thermocycler as follows: 2 min at 94°C, followed by 40 cycles of 94°C for 20 s, 57°C for 20 s, and 72°C for 40 s. The amplified products were resolved on 1.0% agarose gels, stained with Gel Red (Uniscience, Brazil), and visualised using an Image Quant 300 (GE Life Sciences, USA).

### Quantitative RT-PCR

Quantitative RT-PCR (qRT-PCR) was performed using a StepOne Real-Time PCR System (Applied Biosystems, USA) in 96-well optical reaction plates (Applied Biosystems, USA). One microliter of cDNA template, 0.6 μM of each specific primer (Table 1), and 1 μl of Maxima SYBR Green (Thermo Scientific, USA) were used for each reaction. The thermal cycling program was: 10 min at 95°C, followed by 40 cycles of 95°C for 15 s, 57°C for 1 min, and 60°C for 1 min. The threshold cycle was normalised according to the rp49 ribosomal protein expression, and relative gene expression was calculated by the 2^-ΔΔC^T method [38], using expression data generated from adult females fed on sucrose as a reference condition. All depicted data correspond to three independent biological samples analysed in triplicate.

### Sequencing and analysis

Partial fragments amplified from genomic DNA and cDNA for all the analysed genes were sequenced on both strands with BigDye Terminator v3.1 (Applied Biosystems, USA) on an automatic sequencer model ABI 3100 (Applied Biosystems, USA). The resulting sequences were subjected to BLASTn searches using the major databases, NCBI and VectorBase. The sequence analyses after sequencing were performed using Bioedit (http://www.mbio.ncsu.edu/bioedit/bioedit.html [39] and aligned by ClustalW (http://www.ebi.ac.uk/Tools/msa/clustalw2 [40]) software. Signal peptides were predicted by the *SignalP 4.0* algorithm (http://www.cbs.dtu.dk/services/SignalP/ [41]). The phylogenetic tree was constructed using maximum likelihood bootstrap with 1000 replicates, built in the MEGA 6 program [42].

### Determination of cathepsin B activity during vitellogenesis

Three pairs of ovaries dissected from females fed on sucrose (SUC) and at 12, 24, 36, 48, 60, 72, and 84 hours post blood meal (PBM) were homogenised in 60 μl of PBS in the absence of protease inhibitors. Enzymatic activity was measured using 10 μl of homogenate as described above.

### Statistical analysis

All experiments were repeated three times and the data were analysed using one-way or two-way analysis of variance (ANOVA) and the Tukey's HSD (Honestly Significant Difference) test with confidence intervals of 95% with the GraphPad InStat 3.02 software package (GraphPad Software Inc., USA). Experimental values were obtained from three independent assays and expressed as the means ± standard error.

### Results and Discussion

Enzymatic activity profile of cathepsin B

The activity of cathepsin B has been previously characterised by hydrolysis of the specific synthetic substrate, Z-Arg-Arg-NHMec [43], at an acidic pH in ovaries and eggs of Diptera such as *Drosophila melanogaster* [8], *Musca domestica* [9,10] and *Aedes aegypti* [11]. For all of these insects, cathepsin B has been shown to play a role in the degradation of yolk during embryogenesis.

**Fig 1:**
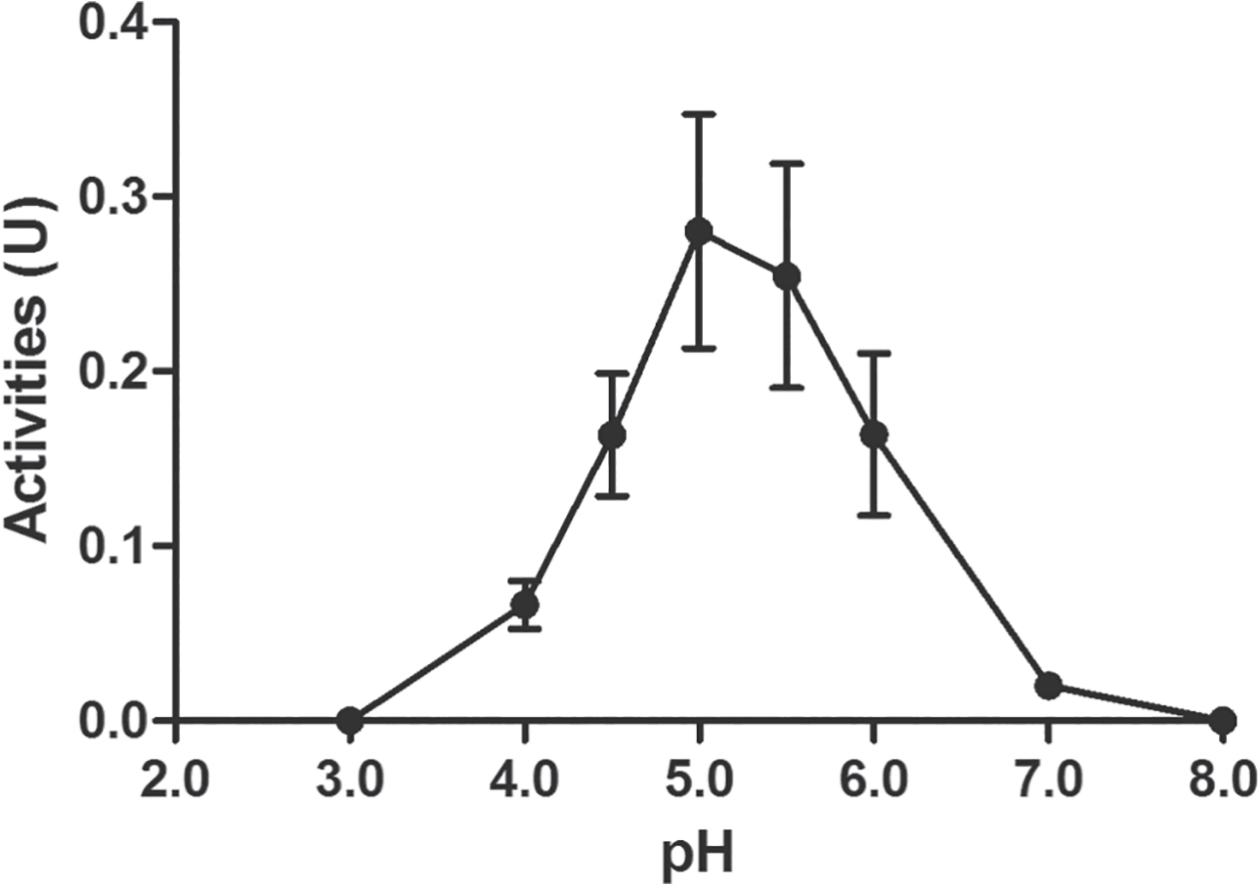
Determination of optimal pH for enzyme activation. Enzymatic activity of total extract of dark eggs (approximately 24 h after oviposition) of *Cx. quinquefasciatus* on Z-Arg-Arg-NHMec was assessed at different pHs at 27°C. The proteolytic activities are expressed in units (U) defined as the amount of enzyme that hydrolyses 1 μmol of substrate per minute. Each point represents the mean expression ± standard error of three independent biological experiments.

Based on these data, we confirmed cathepsin B activity in total extract from dark eggs (approximately 24 h after oviposition) of *Cx. quinquefasciatus* with Z-Arg-Arg-NHMec at an optimum pH level between 5.0 and 5.5 (Fig. 1), similar to the conditions described previously [8–11]. Based on these data, all future experiments were conducted at pH 5.0 at 27°C.

Self-proteolysis

**Fig 2:**
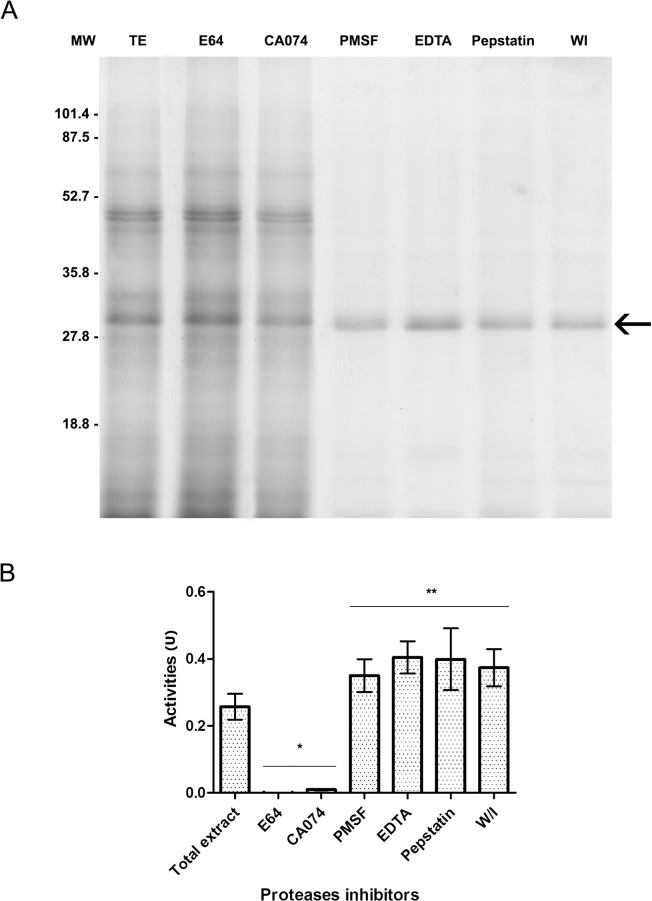
Enzymatic activity analysis of the total extract of *Cx. quinquefasciatus* dark eggs. **A**: 12% SDS-PAGE of the total extract (TE) before or after incubation at 27°C for 18 h without protease inhibitors (WI) or in the presence of specific inhibitors of various proteases: 50 mM E-64 (cysteine proteases); 1 mM CA-074 (cathepsin B); 1 mM PMSF (serine proteases); 2 mM EDTA (metalloproteases); 10 mM pepstatin (aspartyl proteases); WI: without inhibitor. A total of 2 μg of protein was loaded into each lane, and the molecular weight (MW) is shown in kiloDaltons. The arrow points to the approximately 30 kDa band, which was analysed by mass spectrometry. **B**: The proteolytic activity, calculated at pH 5.0, of the total extract from *Cx. quinquefasciatus* dark eggs prior to and after incubation in the presence or not of the same specific protease inhibitors. Proteolytic activity is expressed in units (U) defined as the amount of enzyme that hydrolyses 1 μmol of substrate per minute. Each column represents the mean expression ± standard error of three independent biological experiments; asterisks denote statistically significant divergences compared to the standard condition (total extract) as determined by one-way ANOVA (*: *p* < 0.001; **: *p* < 0.0001) and the Tukey's HSD (Honestly Significant Difference) test.

The SDS-PAGE profile of the dark eggs’ total extract (Fig. 2A) revealed a drastic decrease in band number compared to extracts from vitellogenic ovaries or white eggs (S1 Fig.). These results confirm recent data [44], which describes advanced embryonic development at 24 h after oviposition.

Only one protein band was observed at approximately 30 kDa (the approximate mass of the dipteran cathepsin B homologues previously described [9–11]; Fig. 2A, arrow) within egg extracts that were incubated without protease inhibitors for 18 hours at pH 5.0 and 27°C [10] compared to the total egg extract prior to incubation (Fig. 2A).

Together, these results suggested that at least one active protease remains within the egg extract (Fig. 2B). The residual enzymatic activity was shown to be inhibited by E-64 and CA-074, a specific inhibitor of cysteine proteases [33] and cathepsin B [34], respectively, but not by any other enzyme inhibitors tested, including PMSF (serine proteases), EDTA (metalloproteases) and pepstatin (aspartyl proteases; Fig. 2B).

Mass spectrometry identifies cathepsin B peptides

The 30 kDa band (Fig. 2A) was excised from the gel and submitted for *in gel* reduction, alkylation and trypsinisation. The resulting tryptic peptides were individually analysed by mass spectrometry. Sequest analysis [45] of MS/MS data revealed that the peptides corresponding to the protein band shared identity with two cathepsins B of *Cx. quinquefasciatus* deposited in the VectorBase database with codes CPIJ015761 and CPIJ015762 (S1 Table). In this work, we will refer to CPIJ015761 as CatB1 and CPIJ015762 as CatB2.

The protein sequences of CatB1 and CatB2 identified by peptide analysis were found to display high alignment similarity with a lysosomal cathepsin B of *Homo sapiens* (AAH10240.1) [43] and with a cathepsin B of *Ae. aegypti* (AAEL007585) [11,12] (S2 Fig.; S2 Table). This analysis also identified the catalytic dyad, which consists of a cysteine and histidine residue and is specific to cysteine proteases [43].

The alignment of the deduced amino acid sequences of CatB1 (342 aa) and CatB2 (353 aa) revealed approximately 66% shared similarity (S2 Fig.; S2 Table).

The nucleotide sequence analysis of the two *Cx. quinquefasciatus* cathepsins B genes obtained from the VectorBase database showed 73% shared identity, with the exception of non-coding regions within the 3' UTR region (S3 Fig.), which allowed for the design and synthesis of specific reverse primers for each gene (Table 1). Oligonucleotide specificity was confirmed by sequencing PCR products and by the dissociation curve analysis generated by real-time PCR, which revealed a single peak for each cathepsin B transcript (data not shown).

Expression profiling of cathepsins B1 and B2

Previous work, performed in several insect species, has highlighted the role of a single acting vitellolytic cathepsin B in yolk protein degradation. However, it remains unclear whether multiple peptidases may contribute to targeted protein degradation within the oocyte. In support of potential enzyme cooperativity, Price and co-workers [12] reported the e transcription of four genes of cathepsin B within the fat body of *Ae. aegypti* female following a blood meal; however, the enzymatic activities were not analysed. In contrast, Snigirevskaya and colleagues [46] reported only a single cathepsin B at the yolk granule periphery in the oocytes of the same mosquito. As vitellogenin, this enzyme is synthesised by the vitellogenic fat body as a 44 kDa precursor protein, which is then secreted into the haemolymph and internalised by the developing oocytes. Following fertilisation, it is activated by the acidification of the granule to produce an active enzyme of 33 kDa [11,46].

A single cathepsin B was also described in other dipterans as an enzyme of 39 kDa in *Drosophila melanogaster* [8], 41 kDa in *Musca domestica* [9,10], and 30 kDa in *Blatella germanica* (Dictyoptera) [22] and *Bombyx mori* (Lepidoptera) [16].

**Fig 3:**
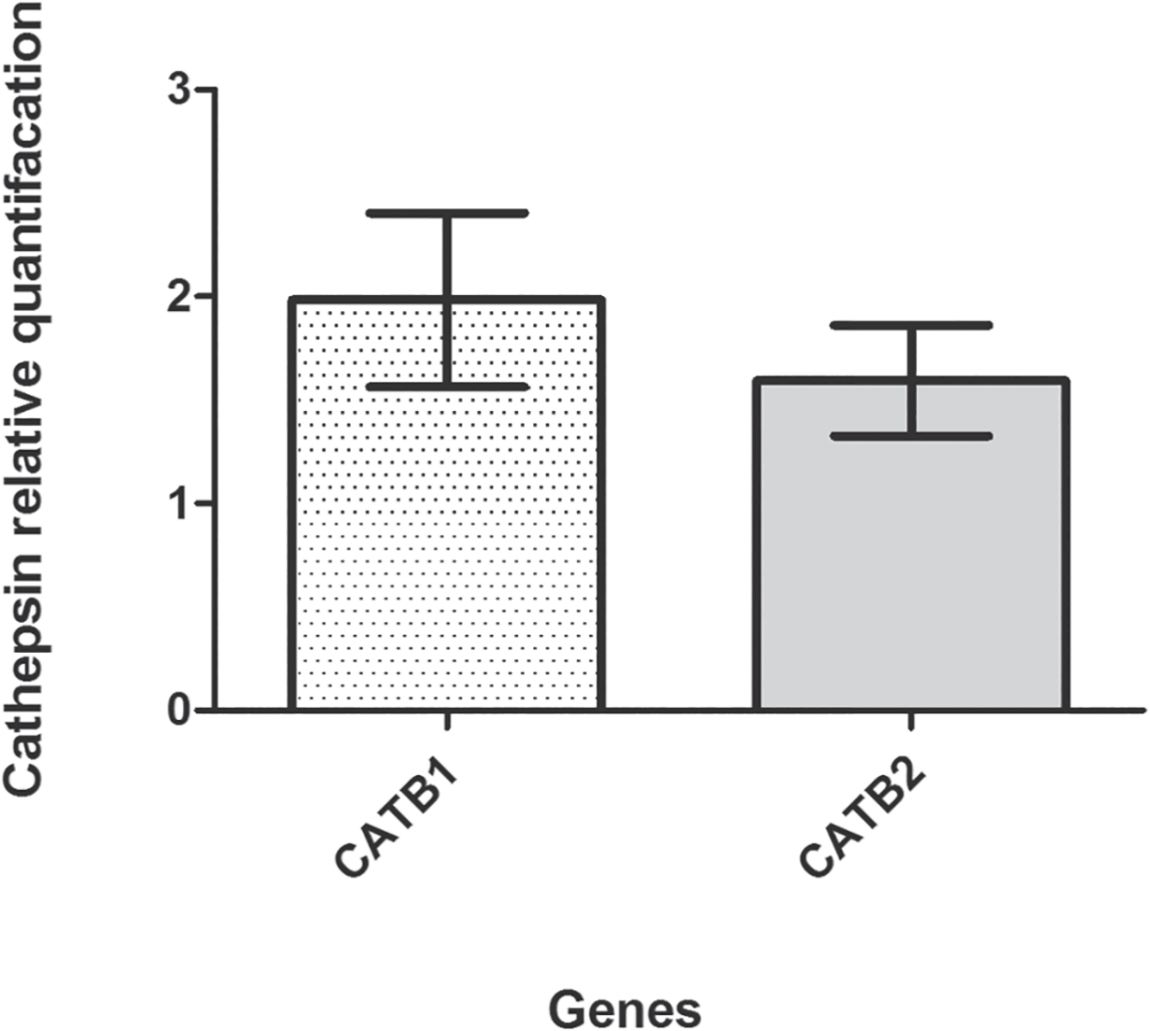
Expression profiling of CatB1 and CatB2 in individual *Cx. quinquefasciatus* females. Quantitative relative real-time PCR was used to examine the transcript levels of CatB1 (dotted bars) and CatB2 (grey bars) from 9 individual *Cx. quinquefasciatus* females 24 h PBM. The mean expression ratio between CatB1 and CatB2 is 1.2 in all of the subjects. Each column represents the mean expression ± standard error of the nine individuals.

These findings led us to investigate whether CatB1 and CatB2 of *Cx. quinquefasciatus* are co-expressed. For this purpose, *Cx. quinquefasciatus* females 24 hours PBM were individually tested by quantitative relative real-time PCR and revealed that CatB1 and CatB2 were simultaneously expressed in each mosquito analysed (Fig. 3).

Based on the finding that the cathepsins B1 and B2 of *Cx. quinquefasciatus* were expressed simultaneously and reside within the same phylogenetic tree constructed from the amino acid sequences of cathepsins B of *Cx. quinquefasciatus* and *Ae. aegypti* [11,12,47], we hypothesise that they are likely the product of a successful gene duplication [48,49] (S4 Fig.).

**Fig 4:**
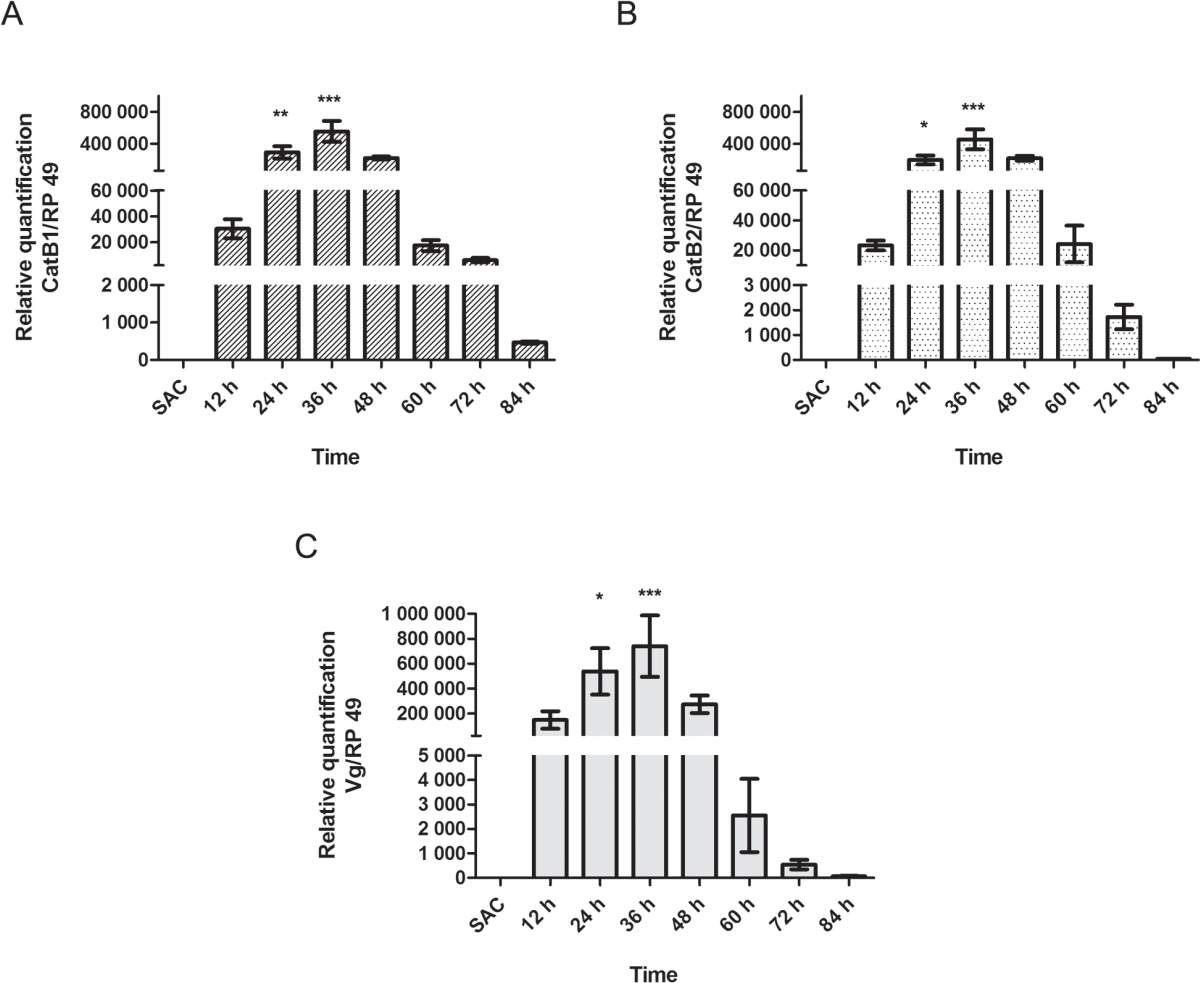
Assessment of vitellogenin and CatB1 and CatB2 transcript levels during *Cx. quinquefasciatus* vitellogenic process. Quantitative real-time PCR was used to examine the transcript levels of CatB1 (A), CatB2 (B), and vitellogenin (Vg; C) of females fed on sucrose (SUC) and every 12 h PBM (12, 24, 36, 60, 72, 84). Each column represents the mean expression ± standard error of three independent biological replicates; asterisks denote data with a statistically significant differences compared to the standard condition (SUC), as determined by one-way ANOVA (*: *p* < 0.05; **: *p* <0.001; ***: *p* < 0.001) and the Tukey's HSD (Honestly Significant Difference) test.

In addition to simultaneous expression, both enzymes were shown to be upregulated post blood meal (Fig. 4), with peak expression occurring at 36 h PBM, similar to vitellogenin (Fig. 4). Interestingly, while the expression of vitellogenin showed a sharp decrease at 60 h PBM, CatB1 and CatB2 expression remained high until 84 h PBM, which coincides with female entry into oviposition (Patricia S. Yogi, unpublished data). The amount of cDNA was confirmed using the RP49 gene, which was also used as housekeeping control gene for the qRT-PCR (S5 Fig.).

Enzyme activity profiling of cathepsins B during vitellogenesis

**Fig 5:**
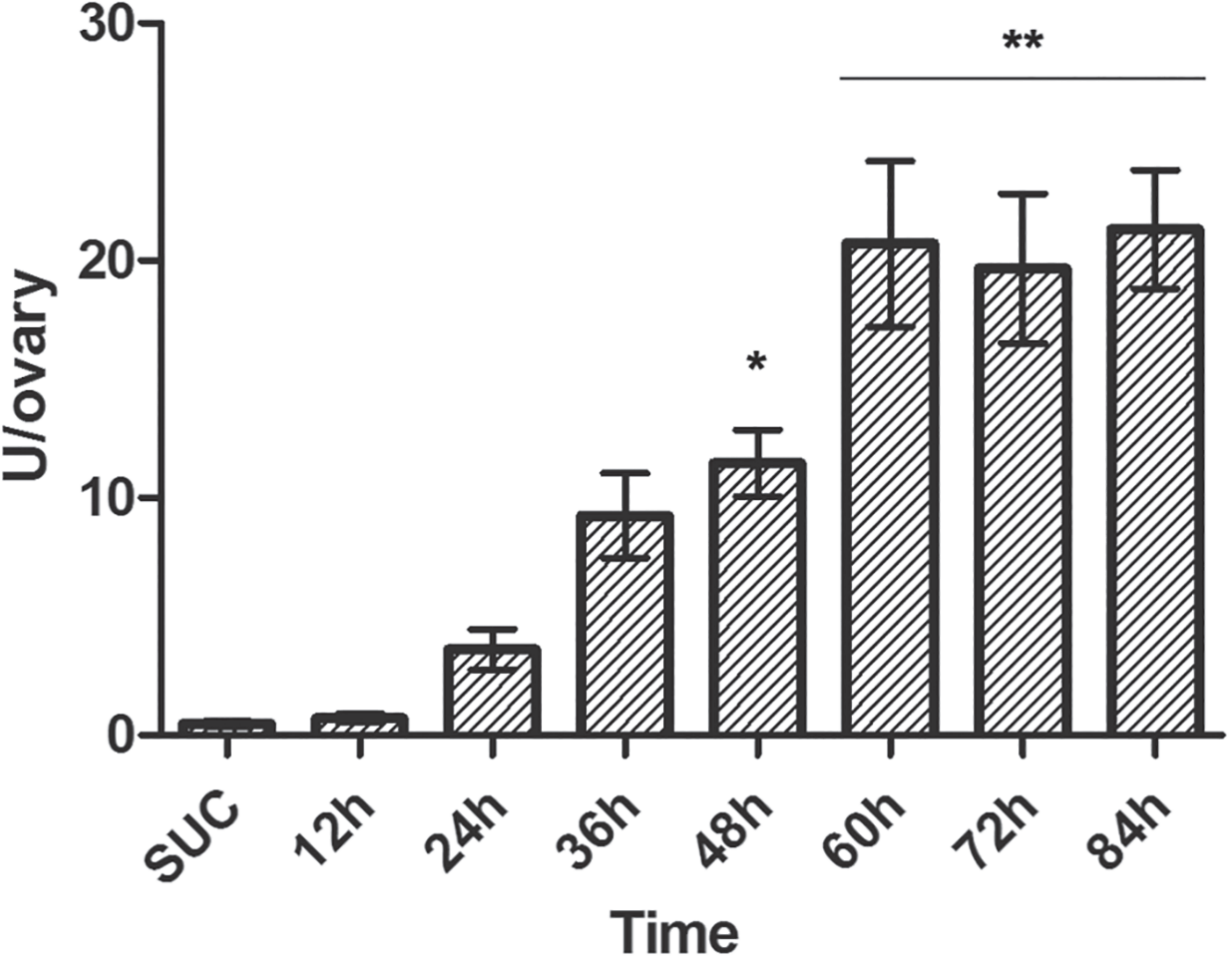
Enzymatic activity profiling throughout the vitellogenic cycle of *Cx. quinquefasciatus*. Enzymatic activity of cathepsin B was measured in a single ovary of adult females fed on sucrose (SUC) and every 12 h PBM (12, 24, 36, 60, 72, 84). U/ovary: Units per ovary, where unit is defined as the amount of enzyme that hydrolyses 1 μmol of substrate per minute. Each column represents the mean expression ± standard error of three independent biological replicates; asterisks denote data with a statistically significant differences compared to the standard condition (SUC), as determined by one-way ANOVA (*: *p* < 0.05; **: *p* < 0.001) and Tukey's HSD (Honestly Significant Difference) test.

While the transcription of both cathepsins B was shown to be upregulated at 12 h PBM (Fig. 4A and 4B), the enzymatic activity was only detectable at 24 h PBM and continuously increased until reaching a plateau at 60 h PBM (Fig. 5). We hypothesise that this observation may be explained by cessation of incorporation, as this time is synonymous with endochorion secretion by the follicular cells [50].

## Conclusions

In this paper, we describe the transcriptional profile of two cathepsin B homologues, which are expressed simultaneously in the fat body of *Cx. quinquefasciatus* vitellogenic females from 12 h PBM.

Both cathepsins were identified by mass spectrometry from the total extract of *Cx. quinquefasciatus* eggs after self- proteolysis. While *in silico* analysis suggested that both sequences harbour catalytic residues that may contribute to the observed enzymatic activity, it was not possible to determine whether both enzymes are simultaneously activated.

Finally, the transcriptional profile of the two cathepsin B genes is similar to that of vitellogenin, which supports the notion that the two enzymes likely cooperate in vitellin degradation.

## Supporting Information

S1 Fig Analysis of the total extract from *Cx. quinquefasciatus* ovaries and eggs. 8% SDS-PAGE was used to visualise the total extract of ovaries (OVA) at 96 and 120 h PBM and eggs 2 h (white eggs) and 24 h (dark eggs) after oviposition. In each lane, 2 g of total protein was loaded, and the molecular weight is shown in kiloDaltons.
(TIF)

S2 Fig Alignment of the deduced amino acid sequences of different cathepsins B. Sequences of *Homo sapiens* (AAH10240.1; NCBI) [43], *Ae. aegypti* (AAEL007585; VectorBase) [11,12] and *Cx. quinquefasciatus* (CatB1: CPIJ015761 and CatB2: CPIJ015762; VectorBase) were compared. Box region: signal peptides; black background indicate identical amino acids; asterisks indicate catalytic site residues: cysteine (C) and histidine (H).
(TIF)

S3 Fig Alignment of the nucleotide sequences of *Cx. quinquefasciatus* cathepsins B. **A**: Alignment of the 3'UTR regions of CatB1 (CPIJ015761) and CatB2 (CPIJ015762) of *Cx. quinquefasciatus*. Boxes indicate the sequences used to design reverse primers. **B**: Alignment of the nucleotide sequences of the *Cx quinquefasciatus* cathepsin B genes: CatB1 (CPIJ015761) and CatB2 (CPIJ015762). Sequences highlighted in black represent conserved nucleotides.
(TIF)

S4 Fig Phylogenetic tree of the cathepsins B. A phylogenetic tree was constructed using the cathepsins B amino acid sequences of *Cx. quinquefasciatus* (CPIJ015761, CPIJ015762; VectorBase) and *Ae. aegypti* (AAEL007585, AAEL007590, AAEL007599; VectorBase).
(TIF)

S5 Fig Cathepsins B and vitellogenin expression during the vitellogenic process of *Cx. quinquefasciatus*. The expression of CatB1, CatB2, vitellogenin (Vg), and ribosomal protein 49 (RP49) was calculated using RT-PCR in females fed on sucrose (SUC) and every 12 h PBM (12, 24, 36, 60, 72, 84). C+: positive control (genomic DNA); C-: negative control (without DNA).
(TIF)

S1 Table The physicochemical properties of the peptide sequences of *Cx. quinquefasciatus* cathepsins B. Listed are each of the peptides detected by mass spectrometry of the 30 kDa band and the percentage of similarity observed between each peptide and the protein sequences of each cathepsin B (CPIJ015761 and CPIJ015762) using BLASTp. The bolded amino acid (H) represents the catalytic histidine. The cross correlation (Xcorr) function was used to assess the quality of peptide spectra matches. The Delta Correlation (DeltaCN) represents the difference between the normalised Xcorrs of the primary and secondary matches.
(DOCX)

S2 Table Comparison of the amino acid sequences of different cathepsins B. The percent of similarity between the amino acid sequences of cathepsins B of *Homo sapiens* (AAH10240.1; NCBI; Barrett and Kirschke, 1980), *Ae. aegypti* (AAEL007585; VectorBase; Cho *et al.*, 1999; Price *et al.*, 2010) and *Cx. quinquefasciatus* (CatB1: CPIJ015761 and CatB2: CPIJ015762; VectorBase) is depicted.
(DOCX)
