# Supplementary figures and images for: Two Cathepsins B Are Responsible for the Yolk Protein Hydrolysis in Culex quinquefasciatus
Source: PLoS One. 2015 Feb 24;10(2):e0118736. doi: 10.1371/journal.pone.0118736 (PMC4339980; doi:10.1371/journal.pone.0118736)

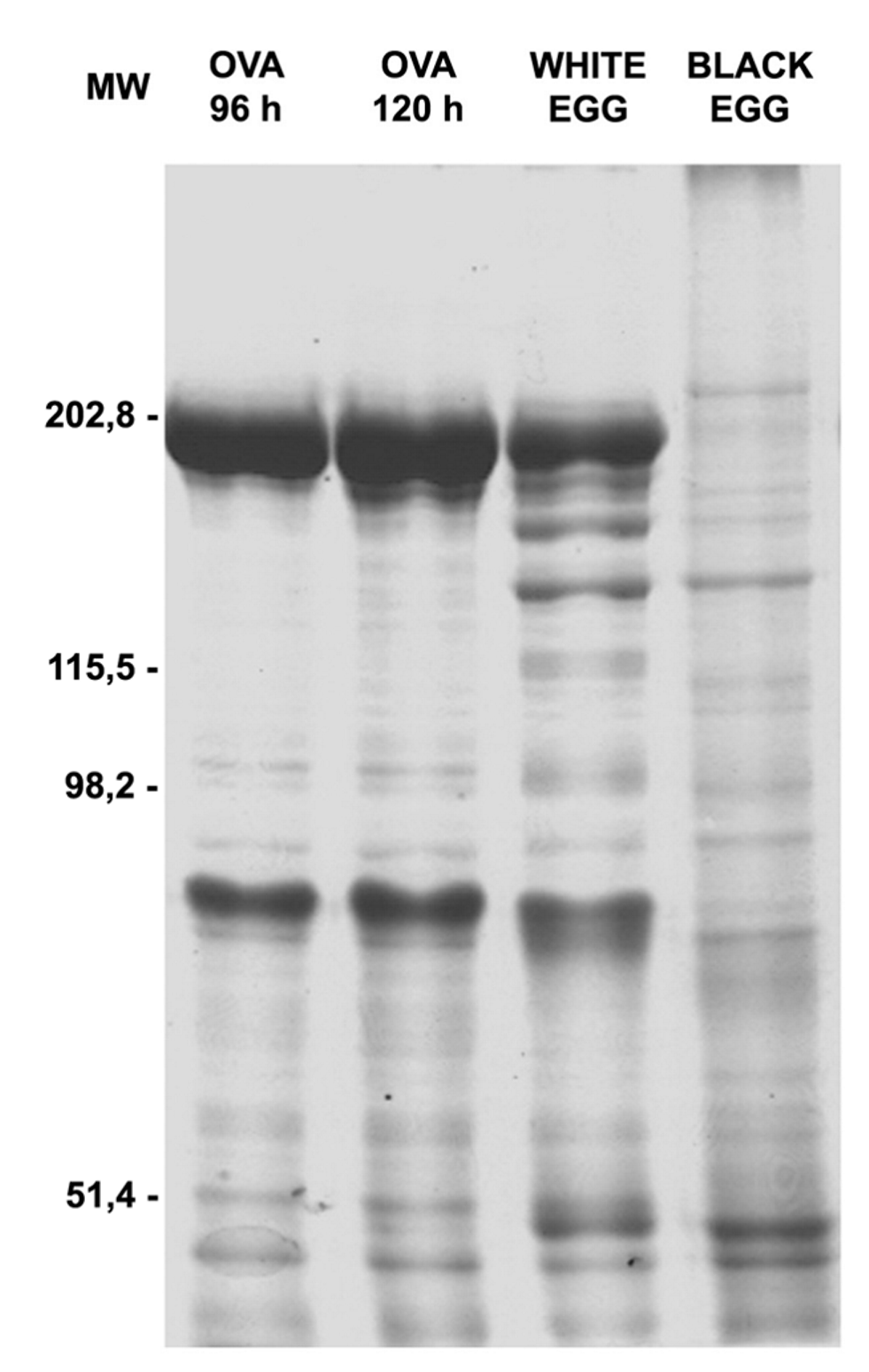

Supplement: S1 Fig — 8% SDS-PAGE was used to visualise the total extract of ovaries (OVA) at 96 and 120 h PBM and eggs 2 h (white eggs) and 24 h (dark eggs) after oviposition. In each lane, 2 g of total protein was loaded, and the molecular weight is shown in kiloDaltons. (TIF) [file pone.0118736.s001.tif]

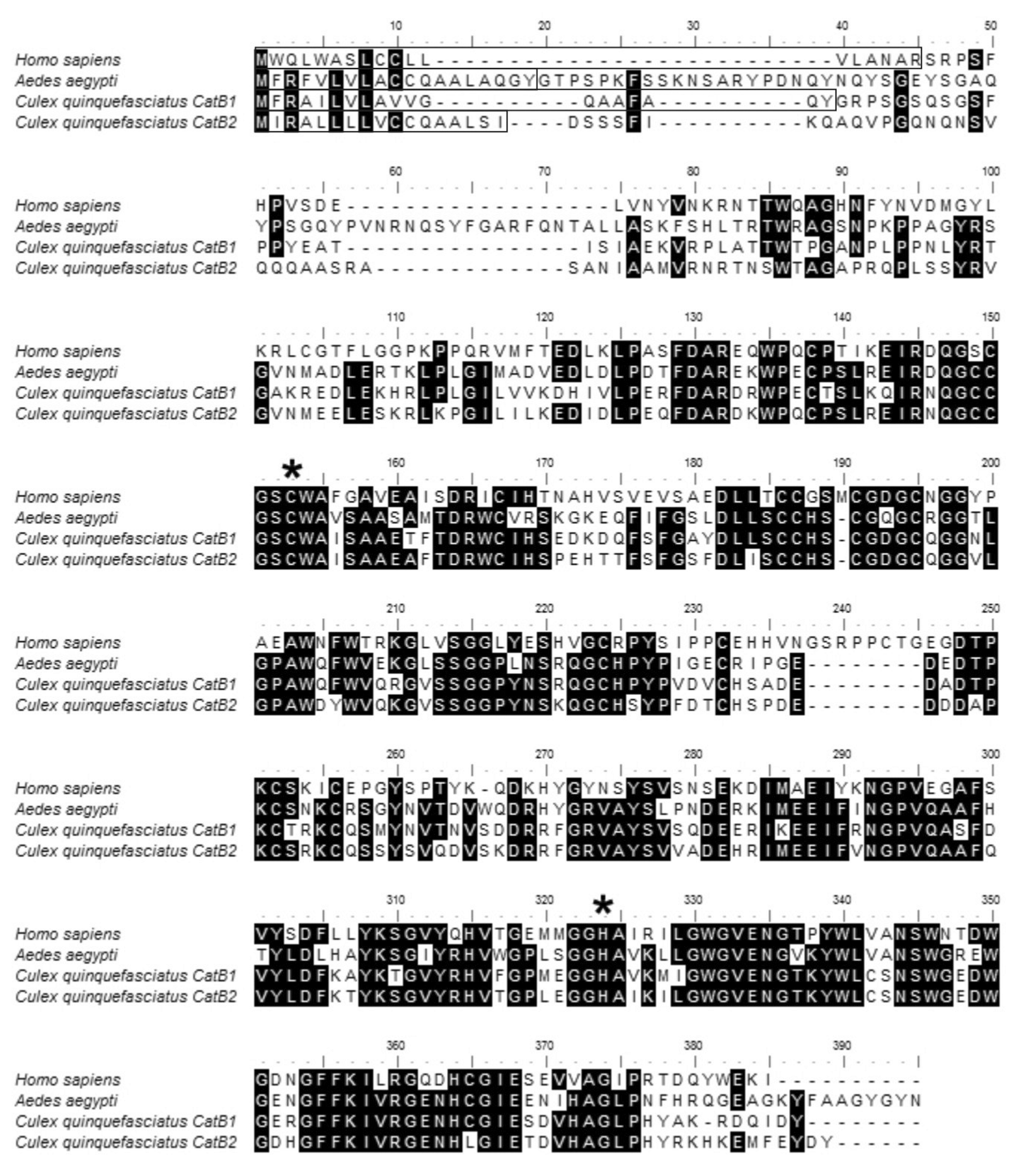

Supplement: S2 Fig — Sequences of Homo sapiens (AAH10240.1; NCBI) [43], Ae. aegypti (AAEL007585; VectorBase) [11,12] and Cx. quinquefasciatus (CatB1: CPIJ015761 and CatB2: CPIJ015762; VectorBase) were compared. Box region: signal peptides; black background indicate identical amino acids; asterisks indicate catalytic site residues: cysteine (C) and histidine (H). (TIF) [file pone.0118736.s002.tif]

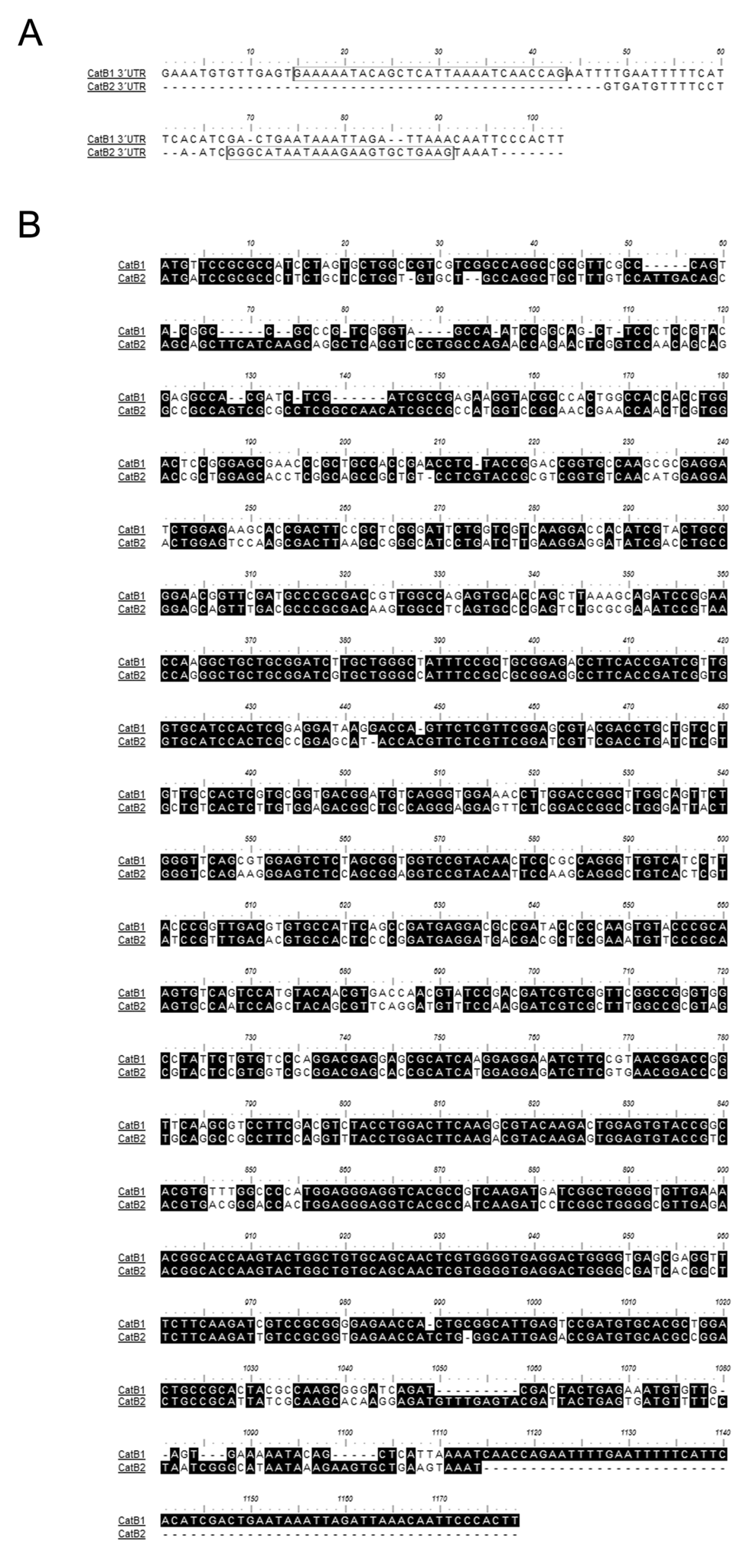

Supplement: S3 Fig — A: Alignment of the 3'UTR regions of CatB1 (CPIJ015761) and CatB2 (CPIJ015762) of Cx. quinquefasciatus. Boxes indicate the sequences used to design reverse primers. B: Alignment of the nucleotide sequences of the Cx quinquefasciatus cathepsin B genes: CatB1 (CPIJ015761) and CatB2 (CPIJ015762). Sequences highlighted in black represent conserved nucleotides. (TIF) [file pone.0118736.s003.tif]

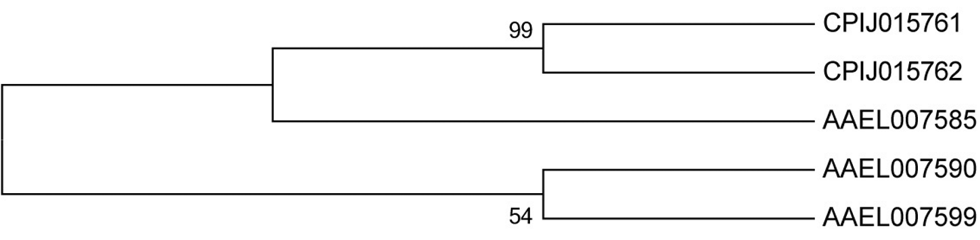

Supplement: S4 Fig — A phylogenetic tree was constructed using the cathepsins B amino acid sequences of Cx. quinquefasciatus (CPIJ015761, CPIJ015762; VectorBase) and Ae. aegypti (AAEL007585, AAEL007590, AAEL007599; VectorBase). (TIF) [file pone.0118736.s004.tif]

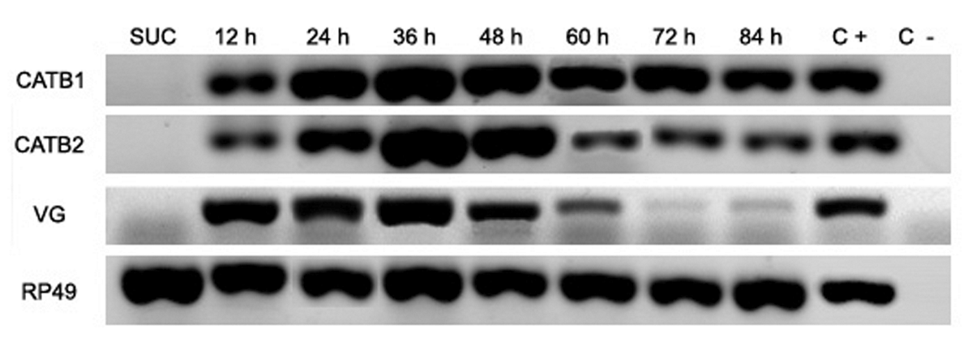

Supplement: S5 Fig — The expression of CatB1, CatB2, vitellogenin (Vg), and ribosomal protein 49 (RP49) was calculated using RT-PCR in females fed on sucrose (SUC) and every 12 h PBM (12, 24, 36, 60, 72, 84). C+: positive control (genomic DNA); C-: negative control (without DNA). (TIF) [file pone.0118736.s005.tif]
