# Supplementary material for: Two Cathepsins B Are Responsible for the Yolk Protein Hydrolysis in Culex quinquefasciatus
Source: PLoS One. 2015 Feb 24;10(2):e0118736. doi: 10.1371/journal.pone.0118736 (PMC4339980; doi:10.1371/journal.pone.0118736)
Supplement: S1 Table — Listed are each of the peptides detected by mass spectrometry of the 30 kDa band and the percentage of similarity observed between each peptide and the protein sequences of each cathepsin B (CPIJ015761 and CPIJ015762) using BLASTp. The bolded amino acid (H) represents the catalytic histidine. The cross correlation (Xcorr) function was used to assess the quality of peptide spectra matches. The Delta Correlation (DeltaCN) represents the difference between the normalised Xcorrs of the primary and secondary matches. (DOCX) [file pone.0118736.s006.docx]

| **Transcripts found** | | **Peptides** | | | **Probability** | | **Score** | **Coverage** | **MW** |
| --- | --- | --- | --- | --- | --- | --- | --- | --- | --- |
| **Name** | **Access number**  **(VectorBase)** | **Sequence** | **CPIJ015761**  **similiraty (%)** | **CPIJ015762**  **similiraty (%)** | **Protein** | **Peptide** | **Xcorr** | **DeltaCn** |  |
| cathepsin B  [*Culex quinquefasciatus*] | CPIJ 015761 |  | | | 5,83E-05 |  | 40,14 | 9,70 | 38160,3 |
|  |  | RVAYSVSQDEERI | 100,0 | 77,0 |  | 5,83E-05 | 2,62 | 0,28 | 595,9 |
|  |  | KDHIVLPERF | 100,0 | - |  | 8,19E-03 | 2,22 | 0,00 | 588,0 |
|  |  | RDRWPECTSLKQ | 100,0 | 58,0 |  | 2,03E-01 | 2,00 | 0,06 | 402,1 |
|  |  | KDHIVLPERFDARD | 100,0 | 71,0 |  | 6,11E-02 | 2,27 | 0,06 | 580,2 |
| cathepsin B  [*Culex quinquefasciatus*] | CPIJ 015762 |  | | | 3,23E-06 |  | 40,15 | 13,30 | 39412,9 |
|  |  | KGVSSGGPYNSKQ | 92,0 | 100,0 |  | 4,22E-02 | 2,71 | 0,22 | 1021,1 |
|  |  | RHVTGPLEGG**H**AIKI | 79,0 | 100,0 |  | 3,94E-05 | 2,81 | 0,29 | 1237,6 |
|  |  | RVAYSVVADEHRI | 77,0 | 100,0 |  | 3,23E-06 | 2,38 | 0,31 | 580,9 |
|  |  | RVAYSVVADEHRI | 77,0 | 100,0 |  | 3,68E-05 | 2,23 | 0,32 | 549,7 |
|  |  | KEDIDLPEQFDARD | - | 100,0 |  | 8,58E-06 | 3,09 | 0,27 | 888,2 |
