# Supplementary material for: Two Cathepsins B Are Responsible for the Yolk Protein Hydrolysis in Culex quinquefasciatus
Source: PLoS One. 2015 Feb 24;10(2):e0118736. doi: 10.1371/journal.pone.0118736 (PMC4339980; doi:10.1371/journal.pone.0118736)
Supplement: S2 Table — The percent of similarity between the amino acid sequences of cathepsins B of Homo sapiens (AAH10240.1; NCBI; Barrett and Kirschke, 1980), Ae. aegypti (AAEL007585; VectorBase; Cho et al., 1999; Price et al., 2010) and Cx. quinquefasciatus (CatB1: CPIJ015761 and CatB2: CPIJ015762; VectorBase) is depicted. (DOCX) [file pone.0118736.s007.docx]

| Species | *H. sapiens* | *Ae. aegypti* | *Cx quinquefasciatus* B1 | *Cx quinquefasciatus* B2 |
| --- | --- | --- | --- | --- |
| *H. sapiens* | - | 45.15 | 43.77 | 44.85 |
| *Ae. aegypti* | 45.15 | - | 60.70 | 58.92 |
| *Cx quinquefasciatus* B1 | 43.77 | 60.70 | - | 66.86 |
| *Cx quinquefasciatus* B2 | 44.85 | 58.92 | 66.86 | - |
